# Autoantibodies as putative biomarkers and triggers of cell dysfunctions in systemic sclerosis

**DOI:** 10.1097/BOR.0000000000001035

**Published:** 2024-08-02

**Authors:** Irene Rosa, Eloisa Romano, Bianca Saveria Fioretto, Mirko Manetti

**Affiliations:** Department of Experimental and Clinical Medicine, University of Florence, Florence, Italy

**Keywords:** autoantibodies, biomarkers, pathogenic mechanisms, scleroderma, systemic sclerosis

## Abstract

**Purpose of review:**

Antinuclear autoantibodies represent a serological hallmark of systemic sclerosis (SSc), with anticentromere, antitopoisomerase-I, and anti-RNA polymerase III antibodies routinely assessed for diagnosis, clinical subset classification, and prognosis. In addition, an increasing number of autoantibodies have been demonstrated to play a pathogenic role by mediating different SSc manifestations. This review aims to give an overview on autoantibodies as putative biomarkers in SSc and discuss their possible pathogenic role as triggers of cell dysfunctions.

**Recent findings:**

Over the years, different autoantibodies have been proposed as biomarkers aiding in diagnosis, disease subtype classification, disease progression prediction, organ involvement, as well as in understanding treatment response. Increasing literature also indicates functional autoantibodies as direct contributors to SSc pathogenesis by exerting agonistic or antagonistic activities on their specific cognate targets.

**Summary:**

In SSc, search and validation of novel autoantibodies with higher diagnostic specificity and more accurate predictive values are increasingly needed for early diagnosis and specific follow-up, and to define the best therapeutic option according to different disease subsets. Moreover, since autoantibodies are also emerging as functional pathogenic players, a better unraveling of their possible pathomechanisms becomes essential to identify new targets and develop promising therapeutic agents able to neutralize their effects.

## INTRODUCTION

Systemic sclerosis (SSc; scleroderma) is an immune-mediated rheumatic disease characterized by a pathogenic triad comprising peripheral microvasculopathy, progressive tissue fibrosis, and autoimmunity. Autoantibodies represent a serological hallmark of SSc, as they are observed in the serum of more than 90% of patients [1–3]. The most relevant SSc-associated autoantibodies are antinuclear autoantibodies (ANA), particularly antitopoisomerase-I antibodies (ATA; anti-Scl70), anticentromere antibodies (ACA), and anti-RNA polymerase III antibodies (ARA), which are routinely assessed for diagnosis, clinical subset classification, and prognosis [1–3]. Other less frequently detected ANA in SSc react with different intracellular targets such as ribonuclear proteins (anti-U1 RNP, anti-U3 RNP/antifibrillarin, and anti-U11/U12 RNP autoantibodies) or nucleolar antigens [anti-Th/To, antinucleolar organizer region 90 (anti-NOR90), anti-Ku, and antipolymyositis/Scl (PM/Scl) autoantibodies] [1–3]. SSc-associated ANA are usually disease specific and mutually exclusive, that is, patients do not switch ANA subset type throughout their disease duration [1–3]. Despite their diagnostic and prognostic value, the role of ANA in SSc pathophysiology has not been completely clarified. On the other hand, other autoantibodies detected in SSc, despite not being useful in clinical daily practice, have been demonstrated to play a pathogenic role by mediating several disease manifestations. The main targets of these so-called functional autoantibodies are represented by cell types, such as endothelial cells (antiendothelial cell antibodies; AECA); cell surface receptors, such as angiotensin II type 1 receptor (anti-AT1R antibodies), endothelin (ET)-1 type A receptor (anti-ETAR antibodies), and platelet-derived growth factor receptor (anti-PDGFR antibodies); and extracellular matrix (ECM) components such as fibrillin or matrix metalloproteinases (MMP) [1,3,4]. The present review aims to give an overview on autoantibodies as putative biomarkers in SSc, and to discuss their possible pathogenic role as triggers of multiple cell dysfunctions. 

**Box 1:**
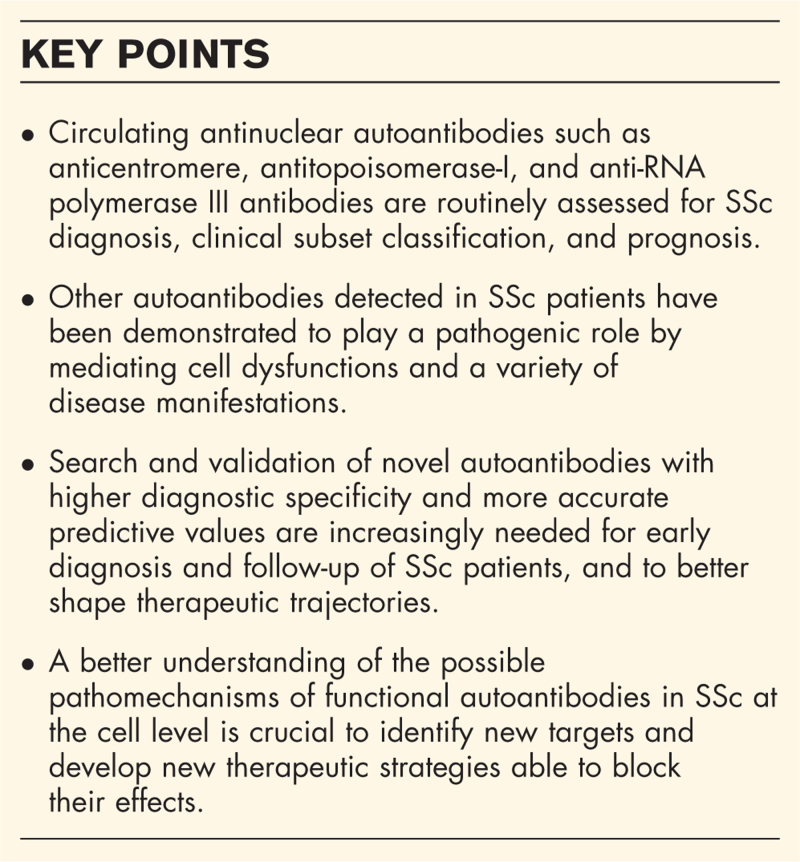
no caption available

## AUTOANTIBODIES AS BYSTANDERS AND BIOMARKERS IN SYSTEMIC SCLEROSIS

ACA, which are mainly directed toward three centromere proteins, namely CENPA, CENPB, and CENPC, are the most frequently observed autoantibodies in SSc patients and are typically associated with the limited cutaneous SSc (lcSSc) subset, featuring long-lasting Raynaud's phenomenon followed by years of progressive thickening of the skin [2,5]. The small percentage of ACA+ patients displaying the diffuse cutaneous SSc (dcSSc) subset is characterized by a lower occurrence of visceral complications and improved survival [6]. ACA+ SSc patients have a higher risk of developing pulmonary arterial hypertension (PAH) [7–9], as well as calcinosis, esophageal dysmotility, and gastrointestinal manifestations [5,8,10]. ACA seropositivity also represents a positive prognostic marker, as patients manifest not only a higher postdiagnosis survival rate but also a lower incidence of scleroderma renal crisis (SRC), cardiac manifestations, and interstitial lung disease (ILD) [5,8]. In a recent study evaluating different ACA isotypes in very early SSc (i.e., patients with ACA-IgG, Raynaud's phenomenon, and/or puffy fingers and/or abnormal nailfold capillaroscopy, but not fulfilling the ACR/EULAR 2013 criteria for SSc) and definite SSc patients, ACA-IgG and ACA-IgM levels were found significantly higher in patients with definite SSc, with progression to established disease being associated with higher IgG isotype at baseline [11]. On these bases, the authors proposed ACA isotype levels as possible biomarkers to identify very early SSc patients at risk for disease progression [11].

Also known as anti-Scl70, ATA represent the second most common ANA in SSc patients and target topoisomerase-I, a nuclear protein that controls DNA tertiary structure during transcription. Usually associated with dcSSc, they have been found not only to correlate with the presence of digital ulcers [9], increased mortality rates in patients with SRC [12], and a higher incidence of ILD [1,5,8,9,13], but also to be a negative prognostic factor in dcSSc patients [8]. Nevertheless, not all ATA+ patients are characterized by a fast evolution of fibrosis, since some of them are only characterized by restrained cutaneous and pulmonary involvement [2,14]. This could be caused by the presence of different ATA isotypes, as SSc patients with both ATA-IgG and ATA-IgM more often experience progression of the disease with respect to those with only ATA-IgG [15]. In a recent study, Liem *et al.*[16] examined the sex-specific risk of ATA on mortality and found that although SSc male patients were more frequently positive for ATA and had an increased mortality rate compared with female patients, such higher mortality was only due to their sex and not to ATA frequency. Conversely, ILD development was found to strongly associate with ATA positivity but not with sex [16,17]. In a Thai SSc cohort of patients, high ATA levels were also recently found to correlate with a short ILD onset, cardiac involvement, and the presence of extensive skin stiffness [18]. Interestingly, when compared to ACA+ SSc patients, ATA+ individuals more frequently showed severe microangiopathy, as assessed by nailfold videocapillaroscopy [19]. In a recent literature study investigating if ATA prevalence in patients with very early SSc could be associated with disease progression, ATA positivity was found to be much lower in very early SSc than in individuals fulfilling the ACR/EULAR 2013 criteria, and ATA-IgG levels were demonstrated to have the tendency to be higher in patients with a more rapid disease progression [20]. Of note, SSc patients with an inverted phenotype, that is, ATA+ but with a lcSSc subset, were reported to be characterized by an increased risk of developing ILD respect to those ACA+ [21]. The presence of ATA has also been found to correlate with a higher risk of cancer [22,23], particularly in the lung in patients with ILD [24]. Finally, circulating B cells from ATA+ SSc patients were seen to abundantly produce both ATA-IgG and ATA-IgA, demonstrating a recent and continuous immune activation that was shown to correlate with the presence and severity of ILD [25].

The presence of ARA is almost exclusive of dcSSc patients and associates with a rapid progression of skin involvement not further evolving after reaching the modified Rodnan skin score (mRSS) peak and, in some cases, also regressing [2,5,26,27]. ARA positivity can also be considered a strong predictor of SRC [1,2,8,12], gastrointestinal manifestations [10], gastric antral vascular ectasia (i.e., a SSc vascular manifestation possibly leading to iron deficiency anemia or acute gastrointestinal bleeding) [2,28], and cancer [1,2,5,29,30]. Interestingly, an association of ARA with silicone breast implant rupture was demonstrated in a multicenter Italian study performed on SSc patients [31]. As far as other clinical associations, ARA are generally not correlated with either SSc-ILD or PAH [2,8]. A recent blood/skin transcriptional and proteomic analysis revealed that different circulating protein markers of fibrosis and different gene expression profiles were displayed by SSc patients positive for ATA or ARA, suggesting that patient stratification according to ANA antibody subtypes may account for different outcomes in clinical trials targeting specific pathogenic mechanisms [27]. A different cell cluster gene expression profile between ATA+ and ARA+ SSc patients was subsequently confirmed by the same group through sc-RNAseq on SSc skin, with transforming growth factor β (TGFβ) ligand-receptor interactions occurring mainly in fibroblasts and smooth muscle cells in early ATA+ dcSSc patients, while mostly in endothelial cells in early ARA+ dcSSc individuals [32^▪^]. In addition, in a study performing gene expression profiling of SSc skin lesions followed by functional enrichment analysis, Inamo identified several pathways that were specifically upregulated according to the type of SSc autoantibody, namely “keratinocyte differentiation” pathways for ACA, “nuclear factor κB signaling” and “cellular response to TGFβ stimulus” pathways for ARA, “interferon α/β signaling” pathways for anti-U1 RNP, and “cellular response to stress” pathways for ATA [33].

Anti-U1 RNP antibodies target the U1 small nuclear ribonucleoprotein (snRNP) complex and are not SSc-specific, as they are usually employed for the diagnosis of mixed connective tissue disease [5]. In a recent monocentric retrospective study, SSc patients positive for anti-U1 RNP were found to be more often of Afro-Caribbean origin and to frequently manifest overlaps with Sjögren's syndrome and/or systemic lupus erythematosus when compared with anti-U1 RNP negative SSc individuals [34]. Moreover, these individuals not only commonly developed ILD, myopathy, and kidney involvement, but also had a worse overall survival [34]. In another study on SSc patients with more than one disease-associated autoantibody type, Clark *et al.*[35] demonstrated that double positivity significantly influences the clinical phenotype of patients. The authors found indeed that, when compared with single positive patients, those with anti-U1 RNP and ATA were younger, commonly showed the dcSSc subset, and had higher rates of overlap features, while those with anti-U1 RNP and ACA had a significantly higher prevalence of PAH and were more frequently affected by myositis [35]. In two other studies, anti-U1 RNP+ SSc patients were also found to be characterized by a higher incidence of SRC or an increased risk of cancer within 2 years from diagnosis [12,22].

Anti-U3 RNP (antifibrillarin) antibodies are directed against a protein of the U3 snRNP complexes called fibrillarin and are detected in 5–14% of SSc patients, where their presence has been associated with the highest incidence of PAH and cardiac involvement [5,8]. Anti-U3 RNP positivity also correlates with severe gastrointestinal involvement and poor prognosis [36]. Although in early SSc, anti-U3 RNP+ patients are characterized by a higher hazard of death, their long-term survival was found to be better compared to anti-U3 RNP negative SSc patients [8].

Anti-U11/U12 RNP antibodies, which target the U11/U12 RNP complex consisting of several proteins involved in alternative mRNA splicing, have been recently demonstrated to associate with the presence of both gastrointestinal manifestations and ILD [37–39]. Moreover, as these antibodies have been found to correlate with an augmented risk of cancer at SSc onset, the possibility of cancer-induced autoimmunity in this subset of patients has been suggested [40].

Anti-Th/To antibodies, directed against numerous proteins of the RNase mitochondrial RNA processing complex, are quite specific for SSc, where their prevalence varies between 1 and 13%, and where they have been associated with lcSSc, ILD, PAH, and SRC [2,5,9,41].

The anti-NOR90 antibodies, which target a 90 kDa nucleolar protein and can be detected in other autoimmune diseases, are present in approximately 5% of SSc patients, where they mostly associate with the lcSSc subset and have a favorable prognosis [2]. In a very recent multicentric cohort study, Dima *et al.*[42] found that anti-NOR90+ SSc patients were more frequently female, had lower mRSS, and lower prevalence of gastrointestinal symptoms respect to anti-NOR90 negative individuals. Anti-NOR90 antibodies were also associated with the presence of SRC, proteinuria, and renal dysfunction [9].

Anti-Ku antibodies, that recognize the Ku complex, that is, a heterodimer involved in DNA repair, transcriptional regulation, and telomere activity, are present in about 2--6% of SSc patients and are commonly associated with the scleroderma-polymyositis overlap syndrome [2].

Anti-PM/Scl antibodies target the human exosome, a macromolecular complex involved in RNA degradation and processing, and are found not only in SSc, but also in other diseases such as polymyositis, dermatomyositis, and scleroderma-polymyositis overlap syndrome [2,43]. In a study performed on a multicenter international cohort of SSc patients, anti-PM/Scl+ subjects were clinically characterized by a higher incidence of myositis, ILD (with a good functional outcome in the first decade of the disease), calcinosis, cutaneous signs of dermatomyositis, and pulmonary fibrosis [2,43,44]. On the contrary, esophageal involvement, PAH, digital ulcers, and cardiac involvement were less frequent [2,43,44]. Anti-PM/Scl+ patients were also found to be at very low risk of death only in the first 10 years of the disease [8]. In another recent study, Richardson *et al.*[45] reported that anti-PM/Scl antibody positivity may confer an increased risk of a heavy burden of calcinosis in SSc patients.

Antibodies to Ro52, a ubiquitous protein over-expressed during inflammation and apoptosis, can be detected in different systemic autoimmune diseases and represent the second most common autoantibody in SSc, overlapping with disease-specific autoantibodies [2]. Several authors demonstrated a significant association between anti-Ro52 positivity and a higher frequency of ILD diagnosis in SSc [2,46,47]. In particular, the presence of anti-Ro52 strongly correlated with pulmonary function loss over time, with the loss rate linearly increasing with anti-Ro52 antibody levels [46]. According to a recent study performed on an Australian cohort of SSc patients, the presence of anti-Ro52 antibodies represents an independent prognostic factor for mortality, and a risk factor for the development of PAH, independently from ILD occurrence [48]. In a recent case series, Kruzer *et al.*[49] characterized ANA+ SSc patients lacking SSc-specific ACA, ATA, and ARA (triple negative), reporting that these individual manifested a high prevalence of anti-Ro52 antibodies, an enrichment for myositis specific antibodies, and an increased risk of ILD. Finally, anti-Ro52 antibodies were found to be more prevalent in cancer-associated SSc [24,29]. Nevertheless, the presence of anti-Ro52, together with anti-U1 RNP or anti-Th/To autoantibodies, was reported to be associated with a decreased risk of cancer when compared with the presence of anti-Ro52 autoantibodies alone [30].

Anticarbamylated protein antibodies (anti-CarPA), that is, antibodies targeting proteins subjected to the posttranslational modification termed carbamylation, which is almost irreversible and can be triggered by inflammation, have been detected in SSc patients, where they have been reported to correlate with the dcSSc subset, ILD, and the presence of digital ulcers [50,51]. No relevant clinical association was conversely found between anti-CarPA positivity and SSc skin involvement assessed with mRSS [52].

Antibodies against vinculin, a protein involved in cell adhesion, were reported not only to be augmented in SSc patients respect to healthy individuals but also to be significantly higher in patients with PAH when compared to those with ILD [53]. Moreover, increased levels of antivinculin autoantibodies were reported to associate with slower gastric transit in SSc patients, thus representing a potential useful marker of SSc-related gastrointestinal manifestations [54,55].

By means of a proteome-wide planar antigen array on a small cohort of SSc patients, antiphosphatidylinositol-5-phosphate 4-kinase type 2 beta (PIP4K2B) and anti-AKT serine/threonine kinase 3 (AKT3) antibodies were found to be the most promising candidates as potential skin and lung fibrosis-associated autoantibodies in SSc [56].

In recent years, numerous novel autoantibodies, including antieukaryotic initiation factor 2B (eIF2B), antiamino acyl-transfer ribonucleic acid synthetase (ARS), and anticyclic citrullinated peptide (CCP) antibodies have been found to be associated with SSc-ILD but, due to their low prevalence, such a clinical association remains to be further validated in larger cohorts of patients [57–59].

In a very recent study, antiinterferon gamma inducible protein 16 (IFI16) autoantibodies were reported to correlate with lcSSc, PAH, and reduced overall survival in patients negative for all SSc-specific autoantibodies [60].

Autoantibodies against annexin V, a calcium-regulated phospholipid-binding protein involved in cell life cycle, exocytosis, and apoptosis, were evaluated in a SSc population at baseline and after a 24-month follow-up and were found not only to be stable overtime but also to be associated with a higher occurrence of PAH and digital microangiopathy [61].

As far as gastrointestinal involvement is concerned, Mc Mahan *et al.*[62] identified antibodies to gephyrin, a protein of the enteric nervous system expressed in human myenteric ganglia, as novel SSc-associated autoantibodies significantly higher among patients with severe constipation, distention, and bloating. In addition, Nakane *et al.*[63] reported a correlation between antiganglionic nicotinic acetylcholine receptor antibodies and various digestive-system problems in SSc patients, such as gastroesophageal reflux, constipation, appetite loss, vomiting, and diarrhea.

In a very recent study employing a global antibody profiling strategy to identify novel antibodies as possible biomarkers in SSc, Liang *et al.*[64] found a significant increase in antiprotein arginine methyltransferase 5 (PRMT5) antibody among SSc patients. These antibodies exhibited strong diagnostic accuracy in distinguishing patients with SSc from healthy individuals, and their titer was also found to correlate with disease progression [64]. Interestingly, immunization of mice with PRMT5 and consequent anti-PRMT5 autoantibody production was shown to induce skin and lung fibrosis [64].

In another study employing a protein array-based approach to identify and validate SSc-specific autoantibodies, antismall nuclear ribonucleoprotein polypeptide A antibodies were proposed as a novel serological biomarker for SSc diagnosis [65]. Moreover, SSc patients with antibodies against Sjögren's syndrome/scleroderma autoantigen 1 were found to manifest an increased risk of cancer compared with negative patients [66].

High levels of anti-AT1R and anti-ETAR antibodies have been reported to be associated with severe SSc vascular manifestations such as digital ulcers and PAH, and to be putative biomarkers to predict the development of such complications and overall mortality [67–69].

The detection of antiphospholipid autoantibodies frequently occurs in SSc, and a significant association of antiβ2 glycoprotein 1 positivity was found with active digital ulcers [70]. Moreover, a higher risk of cardiovascular events was reported in SSc patients positive for antiβ2 glycoprotein 1 and anticardiolipin antibodies [21,71]. A meta-analysis also revealed that antiphospholipid autoantibody positivity correlated with PAH, renal disease, digital ischemia, venous thrombosis, and miscarriage [72,73].

Finally, in an interesting study comparing the performance of three different SSc patient stratification models (i.e., according to LeRoy's cutaneous subtypes, autoantibody profile, or combination of both) in predicting survival, disease progression, and different organ involvement, Elhai *et al.*[74^▪▪^] demonstrated that the antibody-only model outperformed the cutaneous-only one in predicting overall survival, disease progression, and renal and lung fibrosis, while the combined model was more suitable for the prediction of digital ulcers and PAH.

**FIGURE 1:**
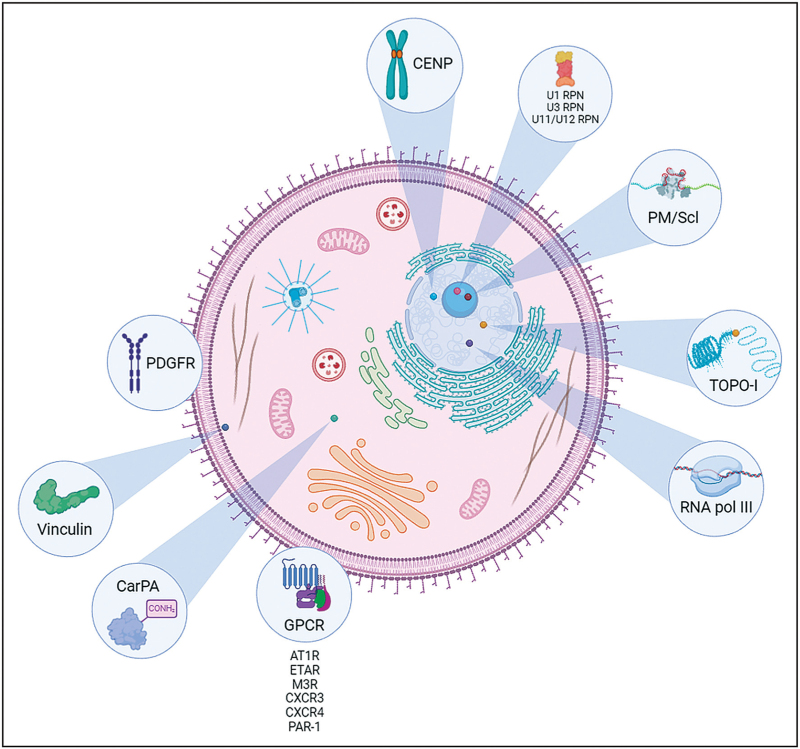
Antigenic targets of the most relevant systemic sclerosis-related autoantibodies. Among antinuclear autoantibodies, anticentromere antibodies are directed toward three centromere proteins, namely CENPA, CENPB, and CENPC; antitopoisomerase-I antibodies target topoisomerase-I (TOPO-I); and anti-RNA polymerase III antibodies bind to RNA polymerase III, the enzyme involved in DNA transcription. Within the nucleolus, anti-U1 RNP, anti-U3 RNP, anti-U11/U12 RNP autoantibodies recognize ribonuclear proteins, while anti-PM/Scl autoantibodies are directed toward a macromolecular complex involved in RNA degradation and processing. The most important plasma membrane and cytoplasmic targets are represented by platelet-derived growth factor receptor (PDGFR); vinculin; carbamylated proteins (CarPA); and several G protein-coupled receptors (GPCR) such as angiotensin II type 1 receptor (AT1R), endothelin-1 type A receptor (ETAR), muscarinic acetylcholine receptor M3 (M3R), C-X-C motif chemokine receptor 3 and 4 (CXCR3, CXCR4), and protease-activated receptor-1 (PAR-1).

A schematic representation of the main antigenic targets of SSc-associated autoantibodies is shown in Fig. 1, whereas a summary of the main associations between autoantibody profile and SSc clinical phenotypes is reported in Table 1.

**Table 1:** Main associations between autoantibody profile and systemic sclerosis clinical phenotypes

Autoantibody	Antigenic target	Association with clinical phenotypes	References
ACA	Centromere proteins CENPA, CENPB, and CENPC	lcSSc; PAH; calcinosis; esophageal dysmotility; GI manifestations; higher postdiagnosis survival rate; lower incidence of SRC, cardiac manifestations, and ILD	[2,5–10]
ATA	Topoisomerase-I	dcSSc; DU; ILD; increased mortality rates in patients with SRC; severe microangiopathy; higher risk of cancer	[1,2,5,8,9,12,13,15–23]
ARA	RNA polymerase III	dcSSc; SRC; GI manifestations; gastric antral vascular ectasia; cancer	[1,2,5,8,10,12,26–30]
Anti-U1 RNP	U1 RNP	ILD; myopathy; kidney involvement; worse overall survival; SRC; increased risk of cancer	[12,22,34]
Anti-U3 RNP	U3 RNP/Fibrillarin	PAH; cardiac involvement; severe GI involvement; poor prognosis	[5,8,36]
Anti-U11/U12 RNP	U11/U12 RNP	GI manifestations; ILD; cancer	[37,38,39,40]
Anti-Th/To	Proteins of the RNase MRP complex	lcSSc; ILD; PAH; SRC	[2,5,9,41]
Anti-NOR90	90 kDa nucleolar protein	lcSSc; SRC; renal dysfunction; lower mRSS; lower prevalence of GI symptoms	[2,9,42]
Anti-Ku	Ku complex	Scleroderma-polymyositis overlap syndrome	[2]
Anti-PM/Scl	Human exosome	myositis; ILD; calcinosis; dermatomyositis; pulmonary fibrosis; calcinosis	[2,43,44,45]
Anti-Ro52	Ro52	ILD; PAH; cancer	[2,24,29,46,47,48,49]
Anti-CarPA	Carbamylated proteins	dcSSc; ILD; DU	[50,51]
Antivinculin	Vinculin	PAH; GI manifestations	[53–55]
Anti-PIP4K2B and anti-AKT3	PIP4K2B and AKT3	Skin and lung fibrosis	[56]
AntieIF2B, anti-ARS, and anti-CCP	eIF2B, ARS, and CCP	ILD	[57–59]
Anti-IFI16	IFI16	lcSSc; PAH	[60]
Antiannexin V	Annexin V	PAH; digital microangiopathy	[61]
Antigephyrin and antigAChR	Gephyrin and gAChR	GI symptoms	[62,63]
Anti-AT1R and anti-ETAR	AT1R and ETAR	DU; PAH	[67–69]
Antiphospholipids	β2 glycoprotein 1 and cardiolipin	DU; cardiovascular events; PAH; renal disease; digital ischemia; thrombosis	[21,70–72]

ACA, anticentromere antibodies; AKT3, AKT serine/threonine kinase 3; ARA, anti-RNA polymerase III antibodies; ARS, amino acyl-transfer ribonucleic acid synthetase; AT1R, angiotensin II type 1 receptor; ATA, antitopoisomerase-I antibodies; CarPA, carbamylated protein; CCP, cyclic citrullinated peptide; dcSSc, diffuse cutaneous SSc; DU, digital ulcers; eIF2B, eukaryotic initiation factor 2B; ETAR, endothelin-1 type A receptor; gAChR, ganglionic nicotinic acetylcholine receptor; GI, gastrointestinal; IFI16, interferon gamma inducible protein 16; ILD, interstitial lung disease; lcSSc, limited cutaneous SSc; MRP, mitochondrial RNA processing; NOR90, nucleolar organizer region 90; PAH, pulmonary arterial hypertension; PIP4K2B, phosphatidylinositol-5-phosphate 4-kinase type 2 beta; PM/Scl, polymyositis/Scl; RNP, nuclear ribonucleoprotein; SRC, scleroderma renal crisis; SSc, systemic sclerosis.

## AUTOANTIBODIES AS PATHOGENIC PLAYERS IN SYSTEMIC SCLEROSIS

Apart from representing relevant disease biomarkers, increasing evidence also suggests a direct pathogenic role of autoantibodies in SSc [3,4,75–77]. Autoantibodies can be defined as pathogenic when they contribute to the development of a disease by mediating its organ and/or systemic manifestations. In particular, functional autoantibodies exert their pathogenic function by directly activating (agonistic effect) or inhibiting (antagonistic effect) a specific molecular pathway through the binding to their cognate autoantigen. In particular, functional autoantibodies can be classified into six different categories based on their mechanism of action, namely activation of target receptors, blockade of target receptors, induction of receptor internalization, neutralization of target ligands, neutralization of other soluble extracellular antigens, and disruption of protein-protein interaction [78].

**FIGURE 2:**
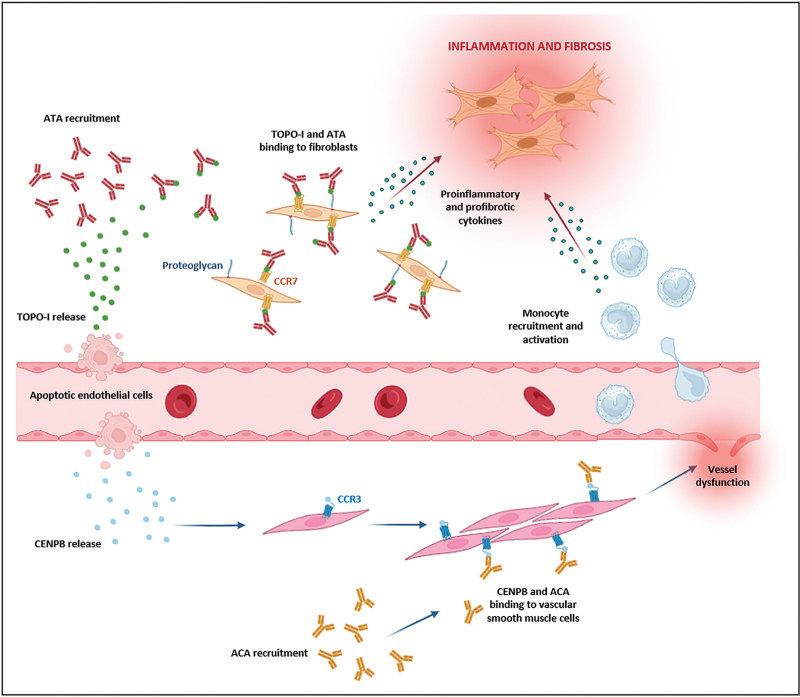
Pathogenic mechanisms of action of antitopoisomerase-I antibodies and anticentromere antibodies. After the release of topoisomerase-I (TOPO-I) from apoptotic endothelial cells and its subsequent binding to the fibroblast membrane, ATA can be recruited on the cellular surface of fibroblasts, possibly via CCR7 or proteoglycans, where they lead to increased monocyte adhesion and activation, ultimately resulting in the release of both proinflammatory and profibrotic cytokines. Similarly, after the release of CENPB by apoptotic endothelial cells, ACA leads to vascular complications by inhibiting CCR3/CENPB-mediated vascular repair on vascular smooth muscle cells.

ATA have been suggested to be pathogenic mainly by promoting incessant inflammation and chronic fibrosis. Indeed, it has been reported that, after the release of topoisomerase-I from apoptotic endothelial cells and its subsequent binding to the fibroblast membrane, ATA can be recruited on the cellular surface of fibroblasts, possibly via CCR7 or proteoglycans, where they lead to increased monocyte adhesion and activation, ultimately resulting in the release of both proinflammatory and profibrotic cytokines [75–77,79] (Fig. 2). Interestingly, in a very recent work, May *et al.*[80^▪▪^] have demonstrated for the first time that ATA are also able to penetrate live cells and localize in the nucleus, where they exert direct effects on intranuclear processes, in particular causing a dose-dependent inhibition of topoisomerase-I activity.

As far as ACA are concerned, it has been proposed that these autoantibodies lead to vascular complications by inhibiting CCR3/CENPB-mediated vascular repair on vascular smooth muscle cells [75,77,79] (Fig. 2). Further evidence of the pathogenicity of both ATA and ACA also derives from the fact that in-vitro stimulation of human dermal fibroblasts with such antibodies can induce a significant increase in profibrotic markers and apoptosis resistance [81], and that incubation of calf pulmonary arterial endothelial cells with SSc sera containing ACA and ATA is able to accelerate endothelial cell aging [82].

A possible pathogenic role of ARA has been proposed on the basis of a study reporting that ARA+ SSc patients with cancer were characterized by a missense mutation in the gene encoding the RPC1 subunit of RNA polymerase III, with the production of an altered protein that, once recognized by the patient's immune system, was able to induce a cross-reactive immune response against the normal RPC1 protein, thereby contributing to the development of SSc [75].

Interestingly, immune complexes purified from the sera of SSc patients bearing different autoantibody specificities (ACA, ATA, ARA, or anti-Th/To) were found to induce proinflammatory and profibrotic pathways in healthy fibroblasts and human umbilical vein endothelial cells, presumably via toll-like receptors [83].

In the last decade, a growing body of evidence has highlighted that several G protein-coupled receptors (GPCR) are targeted in SSc by functional autoantibodies, whose altered levels, cross-reactivity, and synergistic effects may influence the disease pathogenesis [4,84]. Even if not specific to SSc, autoantibodies reactive to AT1R and ETAR, two vascular GPCR, are present in the majority of patients, where they exert agonistic effects [85]. Their capacity to determine SSc-like lesions was initially tested *in vitro* by using IgG fractions of SSc patients positive for anti-AT1R and anti-ETAR on different cell types [69,86,87]. In human microvascular endothelial cells, both antibodies were found not only to amplify profibrotic TGFβ gene expression [69] but also to cause a reduction in wound repair abilities and to induce the expression of interleukin (IL)-8 and vascular cell adhesion molecule 1, with consequent increased recruitment of inflammatory immune cells [86]. In addition, anti-AT1R and anti-ETAR IgG derived from SSc patients with SRC were found to trigger human microvascular endothelial cell proliferation through the mitogen-activated protein kinase pathway, with the subsequent activation of the E26 transformation-specific-1 transcription factor and the induction of tissue factor promoter [88]. Collectively, these data revealed an unknown connection among anti-AT1R and anti-ETAR IgG-induced intracellular signaling, endothelial cell proliferation, and coagulation in the context of SRC [88]. In healthy human dermal fibroblasts, these autoantibodies were reported to increase type-1 collagen expression [86], while in peripheral blood cells, they were found to induce the expression of different proinflammatory cytokines such as interferon-γ, tumor necrosis factor-α, IL-1, IL-6, IL-8, and IL-17, as well as of chemokine (C-C motif) ligand-18, a marker for alternative monocytic activation [87,89,90]. Potential pathogenic effects of anti-AT1R and anti-ETAR antibodies were also tested *in vivo* by the passive transfer of anti-AT1R and anti-ETAR positive IgG fractions of SSc patients into healthy C57BL6/6J mice [68,86]. Interestingly, when histologically assessed, the animals displayed structural lung alterations such as increased cellular density and interstitial infiltrations [86], as well as inflammatory pulmonary vasculopathy [68]. Moreover, after a single IgG transfer, increased neutrophil count was found in bronchoalveolar lavage fluid of SSc IgG-treated mice when compared with healthy control IgG-treated mice, whereas no differences were observed in the counts for macrophages, lymphocytes, or eosinophils [86]. The ability of anti-AT1R to amplify angiotensin II-mediated effects was confirmed in AT1R-immunized mice that, indeed, developed skin and pulmonary inflammation, as well as dermal fibrosis [91].

Antibodies targeting the muscarinic acetylcholine receptor M3 (M3R), an organ-specific GPCR, have been found to contribute to gastrointestinal dysmotility in SSc by inducing neuropathy through the blockade of cholinergic neurotransmission in early disease stages, and by causing myopathy through the inhibition of acetylcholine action on smooth muscle cells with disease progression [3,4,76,92,93]. Interestingly, the administration of intravenous IgG was demonstrated to improve SSc-related gastrointestinal symptoms presumably by neutralizing anti-M3R antibodies [93–95].

Among the GPCR, C-X-C motif chemokine receptor (CXCR)3 and CXCR4 regulate several cell functions and have been involved in the pathogenesis of fibrosis, with anti-CXCR3 antibodies showing potential agonistic activity [4,77]. Although anti-CXCR3 and/or anti-CXCR4 antibodies have been found to be increased in SSc, with anti-CXCR3 autoantibodies from SSc patients preferentially binding to intracellular epitopes instead of extracellular epitopes as in healthy individuals, their pathological functional activity remains to be elucidated [4,76,77,96].

Antibodies against protease-activated receptor-1 (PAR-1), a GPCR with key roles in regulating the interplay between coagulation and inflammation, have been detected in SSc patients with SRC, where they agonistically activate PAR-1 and engage a signaling cascade that finally induces IL-6 production by endothelial cells [3,4,97].

**FIGURE 3:**
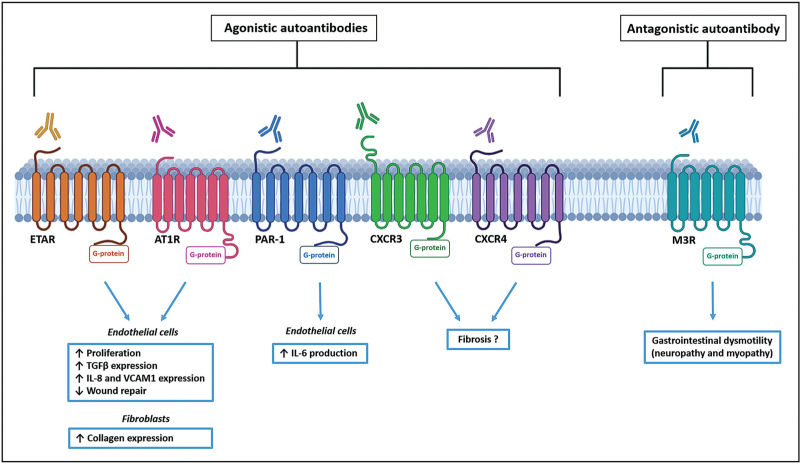
Main pathogenic effects of functional autoantibodies targeting G protein-coupled receptors in systemic sclerosis. Autoantibodies against endothelin-1 type A receptor (ETAR), angiotensin II type 1 receptor (AT1R), protease-activated receptor-1 (PAR-1), C-X-C motif chemokine receptor 3 (CXCR3) and CXCR4 exert agonistic effects, while autoantibodies targeting the muscarinic acetylcholine receptor M3 (M3R) carry out antagonistic activity. IL, interleukin; TGFβ, transforming growth factor β; VCAM1, vascular cell adhesion molecule 1.

Figure 3 shows a schematic representation of the main pathogenic effects of autoantibodies targeting GPCR.

Stimulatory autoantibodies directed against PDGFR, whose activation leads to the induction of proliferation, chemotaxis, and actin reorganization in fibroblasts and smooth muscle cells, have been detected in SSc sera and have been demonstrated to stimulate the conversion of normal fibroblasts into myofibroblasts [78,98,99]. Nevertheless, in the wake of subsequent studies reporting that these antibodies did not exert an agonistic activity and were not SSc-specific [100,101], the authors performed additional experiments and concluded that in the same SSc patient both agonistic and nonagonistic anti-PDGFR autoantibodies may coexist, and that stimulatory autoantibodies can be detected only in SSc but not in healthy individuals or patients with other connective tissue diseases [102,103]. The first indirect evidence of anti-PDGFR antibody pathogenicity was provided by a small study demonstrating that dermal fibroblasts isolated from SSc patients treated with the immunosuppressant rituximab displayed a downregulation of type-1 collagen expression and of the anti-PDGFR autoantibody-triggered intracellular signaling [99,104]. The first direct evidence of anti-PDGFR antibody pathogenicity was instead demonstrated in a skin-humanized severe combined immunodeficiency mouse model engrafted with skin biopsies of healthy donors injected with total IgG purified from the serum of SSc patients [105]. Indeed, mice acquired a cutaneous SSc-like phenotype when injected with pooled SSc IgG as well as with human agonistic anti-PDGFR mAbs obtained from SSc B cells [99,105]. Stimulatory anti-PDGFR autoantibodies from SSc patients have also been demonstrated to induce proliferation and migration of human pulmonary vascular smooth muscle cells *in vitro*[99,106].

In SSc patients, antibodies specific to estrogen receptor-α have been shown to correlate with clinical parameters of disease activity and severity, and with both a higher T cell apoptotic susceptibility and alterations in T regulatory cell homeostasis [107]. Furthermore, anti-CD22 antibodies have been found to reduce the phosphorylation of the inhibitory B cell response regulator CD22, thus stimulating B cell response [108].

Anti-MMP-1 and anti-MMP-3 antibodies were found to inhibit MMP-1 and MMP-3 activity, respectively, suggesting their potential role in impaired ECM turnover and consequent fibrosis development [109,110]. In addition, antifibrillin-1 antibodies purified from the sera of some ethnic groups of SSc patients were demonstrated to activate normal fibroblasts by inducing gene and protein expression of ECM components [111]. However, these latter were not detected in other SSc cohorts [3,112].

Although AECA are not specific to SSc, numerous studies reported their association with disease pulmonary and vascular involvement [3,75]. *In vitro*, AECA have been demonstrated to induce apoptosis in human dermal microvascular endothelial cells and to activate human umbilical vein endothelial cells by upregulating adhesion molecules and increasing interleukin secretion while, *in vivo*, the transfer of these antibodies in a chicken model of SSc resulted mainly in increased endothelial cell apoptosis [3,75].

In a recent study, antibodies targeting skin keratinocytes were detected in SSc circulation and were found to activate human keratinocytes *in vitro* by inducing toll-like receptor-2 and receptor-3 signaling finally leading to IL-1α release, likely contributing to downstream skin fibrosis [113^▪^].

Antibodies targeting telomerase and the shelterin protein telomeric repeat-binding factor 1 were found in an American cohort of SSc patients, where they associated with lung disease and short lymphocyte telomere length, suggesting that these autoantibodies may exert direct pathogenic effects on telomeres [114].

Finally, in a study by Murthy *et al.*[115], IgG serum fraction of SSc patients was found to modulate the secretome of monocyte-like cells toward a proinflammatory and profibrotic activity profile, suggesting their possible direct contribution in regulating immune cell activity.

## CONCLUSION

In SSc, autoantibodies are currently valuable clinical tools routinely used for both diagnosis and disease subtype classification, and may even be prognostic for disease progression, organ involvement, and/or cancer development. Nevertheless, search and validation of novel autoantibodies with higher diagnostic specificity and more accurate predictive values are increasingly needed for early diagnosis, a specific follow-up of patients, and to better define therapeutic choices across the different SSc subsets. Moreover, since autoantibodies are also emerging as functional pathogenic players in SSc, a better unraveling of their possible pathomechanisms becomes essential to both identify new targets and develop promising therapeutic agents able to neutralize their downstream effects at the cell level.

## Acknowledgements

*All of the figures in this study were created in part with BioRender.com.*

### Financial support and sponsorship

*None.*

### Conflicts of interest

*There are no conflicts of interest.*

## REFERENCES AND RECOMMENDED READING

Papers of particular interest, published within the annual period of review, have been highlighted as:

▪ of special interest
▪▪ of outstanding interest
